# Tuberculous spondylitis diagnosed through Xpert MTB/RIF assay in urine: a case report

**DOI:** 10.1186/s12879-016-1844-0

**Published:** 2016-09-26

**Authors:** George Sikalengo, Adria Ramirez, Diana Faini, Kim Mwamelo, Manuel Battegay, Levan Jugheli, Christoph Hatz, Klaus Reither, Emilio Letang

**Affiliations:** 1 Ifakara Health Institute, Ifakara, Tanzania; 2 University Hospital son Espases, Palma de Mallorca, Spain; 3 Division of Infectious Diseases and Hospital Epidemiology, University Hospital and University Basel, Basel, Switzerland; 4 University Basel, Basel, Switzerland; 5 Swiss Tropical and Public Health Institute, Basel, Switzerland; 6 ISGlobal, Barcelona Ctr. Int. Health Res. (CRESIB), Hospital Clínic - Universitat de Barcelona, Barcelona, Spain

**Keywords:** Tuberculosis, Vertebral spondylitis, Xpert MTB/RIF, Pott’s disease, Urine, Case report

## Abstract

**Background:**

Extrapulmonary tuberculosis (EPTB) is associated with high rates of morbidity and mortality. Diagnosis of EPTB is challenging in resource-limited settings due to difficulties in obtaining samples, as well as the paucibacillarity of the specimens. Skeletal tuberculosis accounts for 10–35 % of EPTB cases, with vertebral osteomyelitis (Pott’s disease) representing 50 % of the cases. We present two cases of suspected Pott’s disease, diagnosed through GeneXpert MTB/RIF assay in urine at a rural Tanzanian hospital.

**Case Presentation:**

Case I

A 49-year old male, HIV-1 positive, on co-formulated tenofovir disoproxil fumarate/lamivudine/efavirenz since 2009 and CD4 counts of 205 cells/μL (13 %). He presented with lower back pain and progressive lower limb weakness for two weeks prior to admission. The physical examination revealed bilateral flaccid paraplegia with reduced reflexes, but otherwise unremarkable findings. A lateral lumbar X-ray showed noticeable reduction of intervertebral space between L4 and L5, and a small calcification in the anterior longitudinal ligament between L4 and L5, being compatible with focal spondylosis deformans but inconclusive with regard to tuberculous spondylitis. An abdominal ultrasound showed normal kidneys, bladder and prostate gland. The urinalysis and complete blood counts (CBC) were normal. *M. Tuberculosis* was detected through GeneXpert MTB/RIF in centrifuged urine, with no resistance to rifampicin.

Case II

A 76-year old female, HIV-1 negative, presented with lower back pain and progressive weakness and numbness of the lower limbs for two months prior to admission. The physical examination revealed paraplegia, but otherwise unremarkable findings. The lumbosacral X-ray findings were compatible with spondylosis deformans of the lumbar spine and possible tuberculous spondylitis in L3-L4. The abdominal and renal ultrasound showed normal kidneys and bladder. The urinalysis and CBC were normal. *M. Tuberculosis* was detected through GeneXpert MTB/RIF in centrifuged urine, with no resistance to rifampicin.

**Conclusion:**

We report two cases of suspected tuberculous spondylitis diagnosed through Xpert MTB/RIF in urine samples from a rural Tanzanian hospital. Urine testing using Xpert MTB/RIF reflects disseminated disease and renal involvement, and may offer a feasible additional diagnostic approach for Pott’s disease in rural Africa.

## Background

Extra-pulmonary Tuberculosis (EPTB) is common among people living with HIV/AIDS (PLHIV) in sub-Saharan Africa and is associated with significant morbidity and mortality. Skeletal tuberculosis accounts for 10 to 35 % of all EPTB cases, vertebral osteomyelitis being the most common form accounting for around 50 % of all cases [1]. In resource-limited facilities, diagnosis is difficult and relies on clinical and radiographic findings. This approach has limited diagnostic accuracy and often leads to late diagnosis, with signs of spinal cord compression at admission in 40 to 70 % of cases [2]. Xpert MTB/RIF (Cepheid, Sunnyvale, USA) assay is currently recommended by the WHO for the diagnosis of some forms of EPTB, but this does neither include spinal tuberculosis nor urine testing. Despite previous studies have shown that *M. tuberculosis* DNA can be detected in urine samples from patients with pulmonary TB and EPTB [3], to our knowledge, the diagnosis of Pott’s disease through Xpert MTB/RIF in urine has never been reported to date.

## Case presentation

### Case I

A 49-year-old man living with HIV since 2009 was admitted to our hospital with a complaint of lower back pain for one year. He presented with lower limb weakness for two weeks prior to admission, initially presenting with reduced proximal power in the right lower limb (grade 3/5) and progressive involvement of both lower limbs and development of bilateral flaccid paraplegia. He was on antiretroviral therapy (ART) with co-formulated tenofovir disoproxil fumarate (TDF)/lamivudine (3TC) /efavirenz (EFV), and good adherence. He did not have cough, weight loss or night sweats.

On admission, he was afebrile, and his blood pressure, heart rate, respiratory rate, and oxygen saturation were within normal ranges. The neurological examination revealed no meningeal signs, no cranial nerve palsies, normal strength, sensitivity and reflexes in upper limbs, flaccid paraparesia and reduced reflexes in lower limbs, preserved bowel and bladder habits and no ataxia. He did not have any palpable deformity on his back, and otherwise he had an unremarkable physical examination.

#### Investigations

**Fig. 1:**
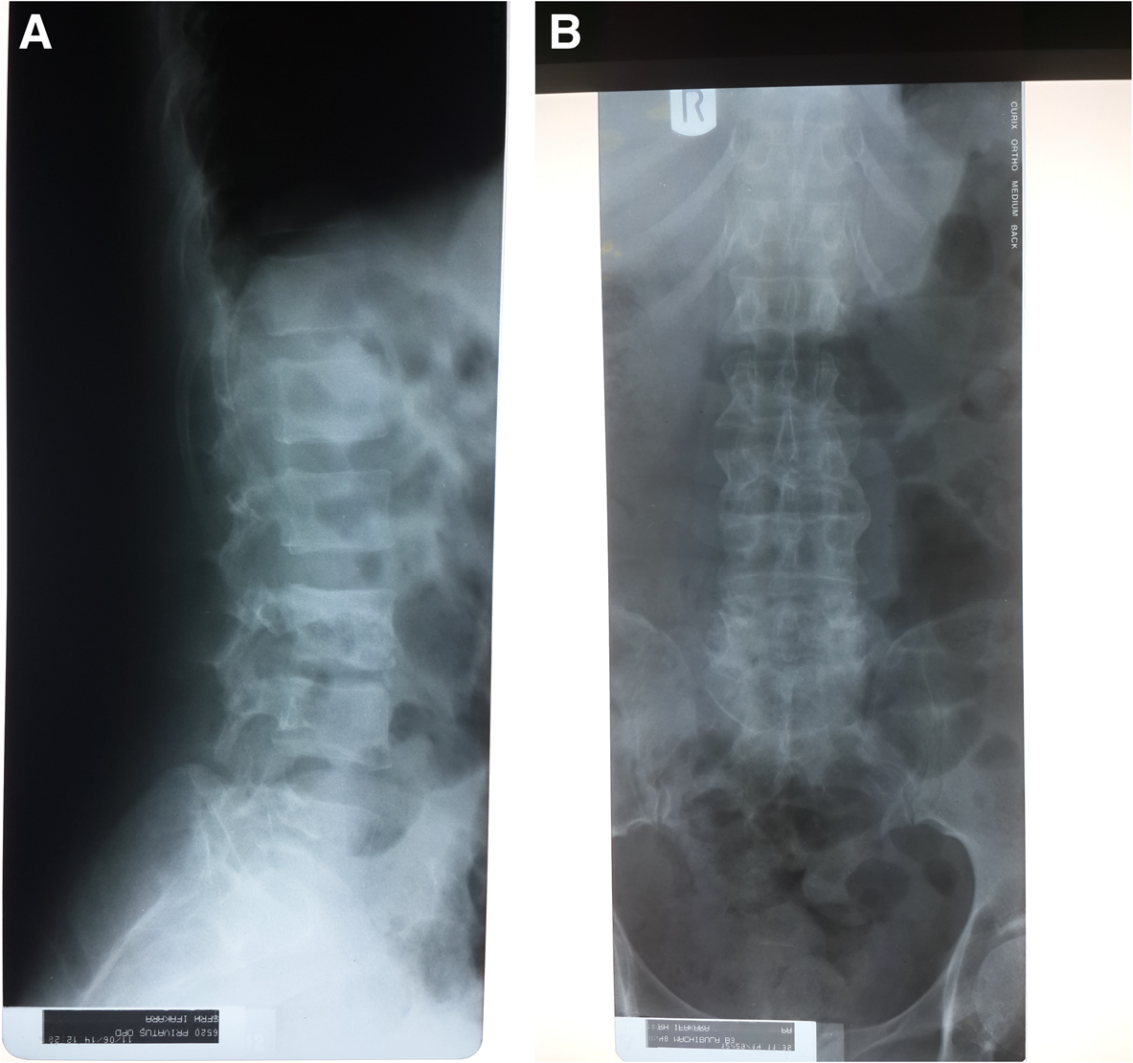
Lumbar Radiographies of Both Patients. **a** Patient 1: Lumbar lateral X-ray showing harmonic lordosis, regular spinal alignment, normal height of vertebral bodies, noticeable reduction of intervertebral space between L4 and L5, small calcification in the anterior longitudinal ligament between L4 and L5, with no obvious destruction of vertebra L4 and L5. **b** Patient 2: Lumbosacral X-ray showing straightening of thoraco-lumbar region, regular spinal alignment, reduced height of vertebra L3 with moderate angulation of the vertebral body, lateral and ventral osteophytes, reduced intervertebral spaces, maximum at L3/L4 and sclerosis of intervertebral joints maximum at L4/L5 and L5/S1

A lateral lumbar X-ray showed harmonic lordosis, regular spinal alignment, normal height of vertebral bodies, noticeable reduction of intervertebral space between L4 and L5, small calcification in the anterior longitudinal ligament between L4 and L5, no obvious destruction of vertebra L4 and L5, being inconclusive with regard to tuberculous spondylitis (Fig. 1a). However, the assessment was limited due to overlaying intestinal gases. The chest radiograph was normal. An abdominal ultrasound showed normal kidneys, bladder and prostate gland. Due to the suspicion of extra-pulmonary TB with spinal involvement, we performed the Xpert MTB/RIF assay in centrifuged urine, which detected *M. Tuberculosis*, with no resistance to rifampicin (Table 1). His CD4 count was 205 cells/μL (13 %), and the complete blood counts (CBC), AST, ALT, creatinine and urinalysis were within normal ranges.

**Table 1:** Showing cycle thresholds (ct) values of each probe for patient 1 and patient 2

	Sample type	MTB Result	RIF Result	Probe D	Probe C	Probe E	Probe B	Probe A	SPC	QC-1	QC-2
Patient 1	Urine	Positive	Susceptible	31.1	29.3	31.8	30.0	30.3	26.4	0.0	0.0
Patient 2	Urine	Positive	Susceptible	34.2	33.3	35.7	33.3	23.3	34.0	0.0	0.0

#### Treatment

Due to the acute nature and progression of the lower limb weakness, the patient was given IV dexamethasone 16 mg in 24 h (4 mg 6hrly in 24 h), with progressive tapering down in 1 week and continuation with oral prednisolone 1.5 mg/kg, tapered down in a period of 4 weeks. Standard antitubercular treatment was started with co-formulated rifampicin 150 mg, isoniazid 75 mg, pyrazinamide 400 mg and ethambutol hydrochloride 275 mg daily for 2 months, to be followed by rifampicin 150 mg and isoniazid 75 mg daily for 4 months adjusted to weight. He continued with his ART regimen, initiating physiotherapy later in the course of treatment.

#### Outcomes

The patient was discharged from hospital upon request from relatives. Despite reporting an initial progressive improvement in power in both lower limbs, he died at home 3 months after starting TB treatment due to non-documented reasons. We could not rule out lack of adherence to anti-tubercular drugs.

### Case II

A 76 year old woman, HIV negative, presented with a 2 month history of lower back pain and a 2 week history of progressive weakness and numbness of the lower limbs without a prior history of trauma. She complained of night sweats and occasional fever without respiratory symptoms. Neurological examination revealed no meningeal signs, no cranial nerve palsies, normal strength, sensitivity and reflexes in upper limbs, flaccid paraplegia and areflexia, preserved bowel and bladder habits and no ataxia. She did not have any palpable deformity on her back, and otherwise, the rest of the physical examination was unremarkable.

#### Investigations

Determine HIV-1/2 (Alere, Waltham, USA) was negative. A lateral lumbosacral X-ray showed reduced height of vertebra L3 with moderate angulation of the vertebral body; lateral and ventral osteophytes; reduced intervertebral spaces, maximum at L3/L4; and sclerosis of intervertebral joints, maximum at L4/L5 and L5/S1. These findings were compatible with spondylosis deformans of lumbar spine and possible additional tuberculous spondylitis in L3–L4 (Fig. 1b). A chest radiograph was not performed. An abdominal and renal ultrasound showed normal kidneys and bladder. The stool analysis, urinalysis and CBC were normal. Due to the suspicion of extra-pulmonary TB with spinal involvement, we performed the Xpert MTB/RIF assay on centrifuged urine, which detected *M. tuberculosis*, with no resistance to rifampicin (Table 1).

#### Treatment

Standard antitubercular treatment was started with co-formulated rifampicin 150 mg, isoniazid 75 mg, pyrazinamide 400 mg and ethambutol hydrochloride 275 mg daily for 2 months, to be followed by rifampicin 150 mg and isoniazid 75 mg daily for 4 months, adjusted to weight. The possibility of transfer for surgical treatment was offered but not possible due to economic constraints.

#### Outcomes

At the time of hospital discharge, she had neither back pain nor more fever or night sweats. She had reduced reflexes without significant changes in power. She attended one physiotherapy visit and never returned to the hospital. She was taken by her relatives to another region, stopped treatment and died at home due to non-documented reasons.

## Differential diagnoses

Tuberculous spondylitis most commonly affects the angles of vertebral bodies and thoracic and lumbar intervertebral joints leading to vertebral narrowing and eventually vertebral collapse. There are a wide number of diseases that can present with low back pain and lower limb weakness, including intervertebral discopathies, malignancies, infections, and inflammatory, vascular, and metabolic causes.

We considered the possibility of herniated disc/spinal stenosis as a possible initial diagnosis. Despite the lack of proper imaging techniques, the physical examination was not compatible. Malignancies like multiple myeloma, spinal cord tumours and other metastases, are in the spectrum of the differential diagnosis,and could not be reliably ruled out with the available imaging techniques and lack of histopathology diagnostic facilities. Transverse myelitis and its etiologies is among the differential diagnosis. Infections associated with transverse myelitis include HIV, HTLV-1, herpes viruses,Lyme disease,mycoplasma, brucella, syphilis, Zika virus, and schistosomiasis. Some of these infections could not be ruled out due to diagnostic limitations, and others (such as syphilis or schistosomiasis) were not assessed after having a positive Xpert MTB/RIF result. Other non-infectious causes of transverse myelitis include multiple sclerosis, acute disseminated encephalomyelitis, neurosarcoidosis, paraneoplastic syndromes, and systemic inflammatory autoimmune disorders such as ankylosing spondilitis, antiphospholipid antibody syndrome, Behçet disease, mixed connective tissue disease, rheumathois arthritis, scleroderma, Sjögren syndrome and systemic lupus erythematosus. None of these diseases was considered due to the lack of associated compatible symptoms. Other infectious causes like bacterial osteomyelitis and epidural abscesses could have caused a similar clinical picture. However, the plain radiographs, although not sensitive enough for diagnosing osteomyelitis, were not compatible. Other diseases such as acute inflammatory demyelinating polyneurolopathy (Guillain-Barré syndrome), vascular myelopathies like anterior spinal artery infarction due to atherosclerosis or embolic disease and metabolic causes like vitamin B12 deficiency should also be considered. Again, these entities could not be reliably ruled out due to diagnostic limitations. However, the chronic clinical evolution is non-compatible with arterial spinal artery infarction; the lack of ascending weakness typically seen in Guillain-Barré syndrome was not observed; and the absence of macrocytic anaemia, although not excluding it, makes the diagnosis of vitamin B12 deficiency unlikely.

To reach a definitive diagnosis, well-equipped microbiology and histopathology laboratories would have been needed, as well as imaging studies like MRI and CT scans. Unfortunately, none of these are available neither at our facility nor at the average rural hospital in Tanzania. In addition, CSF analysis can be important to exclude some of the above pathologies,

Given these diagnostic limitations, a rational workup to diagnose Pott’s disease in the context of compatible symptoms adapted to rural Africa would include: a complete blood count; an erythrocyte sedimentation rate (which can be markedly elevated in Pott’s Disease); a chest radiograph; a dorso-lumbar radiograph (despite radiographic changes associated with Pott’s disease present relatively late and are difficult to interpret by clinicians without training in radiology); and, based on our experience with these two cases, an Xpert MTB/RIF in urine. In the two cases reported, the high specificity and positive predictive value of Gene xpert MTB/RIF assay in the frame of a high prevalence of tuberculosis in our area, we are confident that both patients had tuberculosis.

## Discussion

Tanzania is among the 22 high TB burden countries, which collectively account for more than 80 % of the global burden for tuberculosis. The prevalence of tuberculosis in Tanzania is 295/100,000 people and more prevalent in males than females. Worldwide, EPTB accounts for 25 % of all TB cases, and even higher percentages in HIV-infected individuals, which can represent 15–50 % of the total TB incidence in HIV prevalent settings. In some studies bone and joint TB accounts for around 15–20 % of all EPTB cases in high prevalence settings but the true incidence is still unknown [4]. Existing tests are limited in accuracy and time to diagnosis, and require invasive procedures, which are often not available in rural settings of low-income countries.

The Xpert MTB/RIF assay has been widely implemented in sub-Saharan Africa. However, few data are still available after this implementation. One study conducted in a primary care clinic in rural South Africa has shown that this test could increase the number of patients starting TB treatment and reduce delay in treatment initiation [5]. Neverthelessit does not seem to have an effect in reduction of TB related morbidity [6]. The Xpert MTB/RIF assay is able to detect both the presence of *Mycobacterium tuberculosis complex* DNA and rifampicin drug-resistance in 2 h. The test has excellent accuracy when performed in sputum and was endorsed for the initial diagnosis of TB and multi drug resistant-TB by the WHO in 2010 for this purpose [7]. Information regarding its performance in non-sputum specimens is emerging, but it is not sufficiently validated in HIV-prevalent settings. Recent recommendations from WHO [8] propose Xpert MTB/RIF as a replacement test for specific non-respiratory specimens but not urine, blood or stool samples due to lack of sufficient evidence.

Two studies including a large number of non-respiratory samples including biopsy specimens from tissues, lymph nodes and fine-needle aspirates, pus, urine, gastric aspirates, body fluids including synovial, pericardial, pleural, peritoneal, and cerebrospinal fluid specimens tested with Xpert MTB/RIF demonstrated a high diagnostic accuracy for EPTB. Sensitivity varied widely across different sample types [9, 10]. A meta-analysis evaluating Xpert MTB/RIF performance for EPTB showed a sensitivity of 83.1 % (95 % CI 71.4–90.7 %) in lymph nodes, 80.5 % (95 % CI 59.0–92.2 %) for TB meningitis and 46.4 % (95 % CI 26.3–67.8 %) for pleural fluid [11].

A number of previous studies showed that *M. tuberculosis* DNA can be detected in urine samples. Sensitivity, specificity and techniques used varied between studies. In a study evaluating non-sputum samples for diagnosis of EPTB, six culture confirmed urine samples were included, and *M. Tuberculosis* was detected in all samples by Xpert MTB/RIF [3]. To date, in the largest study including urine samples for diagnosis of EPTB, Xpert MTB/RIF was positive in 11 of 13 positive urine cultures, corresponding to a sensitivity of 87.5 % (95 % CI: 71–104) [10]. In people with unknown HIV status, Xpert MTB/RIF has been shown to have relatively good sensitivity. While the sensitivity reported in HIV infected outpatients prior to ART initiation was 19 % [12], a role for urine testing using Xpert MTB/RIF has been suggested among hospitalized HIV-infected patients, showing a higher diagnostic yield than sputum (64 % vs 27 %) [13–15]. Detection of *M*. tuberculosis in urine reflects disseminated disease but in our case both patients had no leukocituria and had normal abdominal ultrasound scans.

The most common symptom of Pott’s disease is progressively increasing local pain over weeks to months. Constitutional symptoms are present in less than 40 % of cases. Progression of untreated disease may lead to spinal compression and paraplegia [4]. Early diagnosis of Pott’s disease is pivotal to start immediate treatment for TB and improve prognosis. Late presentation may lead to advanced neurological deficits requiring long rehabilitation and carrying a worse prognosis [16]. Performance of Xpert MTB/RIF in tuberculosis spondylitis has been reported in very few studies. In culture and histology confirmed 71 samples from 69 individuals suspected of Pott’s disease, Xpert MTB/RIF had a sensitivity of 95.6 % and specificity of 96.2 % [17]. Diagnosis of tuberculous spondylitis using Xpert MTB/RIF in rural Tanzania has been previously reported in a HIV-negative patient presenting with a paraspinal abscess, and detected in tissue sample through fine needle aspiration [18]. One of the limitations of our report is that the cause of death in both of these cases could not be ascertained, and a definitive diagnosis of Pott’s disease could not be reached. However, both cases were shown to have tuberculosis and were diagnosed with advanced neurological impairment with suggestive radiological findings in one of them. Reactivation of infection with progression to clinical disease can happen in the context of immunosuppression due to several factors. Both patients had some degree of immunosuppression, one due to HIV infection with low CD4 counts and the other one due to old age. We believe that late presentation with advanced disease as well as withdrawal from follow-up led to the poor outcome of both patients.

Surgical treatment is recommended in subjects with Pott’s disease and neurological symptoms. However, good outcomes using a conservative approach with anti-tuberculous treatment only, have also been reported [19]. In the two cases presented, referral to specialized hospitals for surgical treatment was not possible due to economic constrains of the patients.

## Conclusions

The etiologic diagnosis of Pott’s disease remains problematic in rural sub-Saharan Africa, and Xpert MTB/RIF may provide an affordable adjunct diagnostic approach in both HIV and non-HIV patients. We present two cases of Pott’s disease diagnosed through Xpert MTB/RIF in urine samples. To our knowledge, this has never been reported before, and larger studies are needed to assess the feasibility and impact of this approach.

## Abbreviations

ALT: Alanine Aminotransferase
ART: Antiretroviral therapy
AST: Aspartate Aminotransferase
CBC: Complete Blood Count
CD4: *C*luster of differentiation 4
EPTB: Extrapulmonary tuberculosis
HIV: Human Immunodeficiency Virus
HTLV-1: Human T-lymphotropic Virus type 1
PLHIV: People Living with HIV/AIDS
TB: Tuberculosis
WHO: World Health Organization
